# When multi-functional landscape meets Critical Zone science: advancing multi-disciplinary research for sustainable human well-being

**DOI:** 10.1093/nsr/nwy003

**Published:** 2018-01-09

**Authors:** Ying Luo, Yihe Lü, Bojie Fu, Paul Harris, Lianhai Wu, Alexis Comber

**Affiliations:** 1 State Key Laboratory of Urban and Regional Ecology, Research Center for Eco-Environmental Sciences, Chinese Academy of Sciences, Beijing 100085, China; 2 University of Chinese Academy of Sciences, Beijing 100049, China; 3 Joint Center for Global Change Studies, Beijing 100875, China; 4 Rothamsted Research, North Wyke, Okehampton, Devon EX20 2SB, UK; 5 School of Geography, University of Leeds, Leeds LS2 9JT, UK

**Keywords:** multi-functional landscapes, Critical Zone, ecosystem services, sustainable development, human well-being

## Abstract

Environmental degradation has become one of the major obstacles to sustainable development and human well-being internationally. Scientific efforts are being made to understand the mechanism of environmental degradation and sustainability. Critical Zone (CZ) science and research on the multi-functional landscape are emerging fields in Earth science that can contribute to such scientific efforts. This paper reviews the progress, similarities and current status of these two scientific research fields, and identifies a number of opportunities for their synergistic integration through functional and multi-functional approaches, process-based monitoring, mechanistic analyses and dynamic modeling, global long-term and networked monitoring and systematic modeling supported by scaling and deep coupling. These approaches proposed in this paper have the potential to support sustainable human well-being by strengthening a functional orientation that consolidates multi-functional landscape research and CZ science. This is a key challenge for sustainable development and human well-being in the twenty-first century.

## INTRODUCTION

In 2001, the concept of the Critical Zone (CZ) was defined by the US National Research Council (NRC) as a heterogeneous near-Earth surface environment from the top of the vegetation canopy to the bottom of the aquifer that incorporates the near-surface biosphere and atmosphere, the entire pedosphere and the surface and near-surface portion of the hydrosphere and lithosphere [1]. CZ science and theory also encompasses the impacts of anthropogenic activity on Earth surface systems with important implications for sustainable development [2]. By definition, therefore, a comprehensive CZ approach is multi-functional and integrates Earth surface processes at multiple spatial and temporal scales as well as across different gradients (climate, environmental, topographic and anthropogenic) [3]. The cumulative effect of these processes impacts on the mass and energy exchange necessary for biomass productivity, biogeochemical cycling and water storage [4].

CZs have some common characteristics with ecosystems and landscapes. An ecosystem is composed of all living things interacting with each other and their non-living environments—from the local system to the potential dispersal range of all species within this system [5]. Tansley's classical definition of ‘ecosystem’ as ‘one physical system’ is similar to the CZ concept in respect of considering spatiotemporal scales, with most differences in the vertical dimension [6]. Landscapes are composed of different types of ecosystems that represent comprehensively dynamic and heterogeneous Earth surface processes. Landscapes may represent a synthesis of natural, semi-natural and artificial ecosystems and processes on the Earth’s surface, each containing a particular mix of structures and functions [7–12] that may simultaneously support different ecological, economic, social, cultural and aesthetic values [7,13]. Conceptually, ‘ecosystems’ and ‘landscapes’ emphasize the horizontal dimension of the Earth’s surface, as well as the syntheses of structures, processes and functions. However, CZ science (currently) emphasizes *deep* depth, *deep* time and *deep* coupling [14], the first two of which are beyond the scope of consideration by the discipline of ecology in ecosystem and landscape research. The concept of *deep* coupling provides an opportunity to integrate landscape research and CZ science, as both disciplines are interested in consideration of the functional aspects of landscapes and the ability to support human society.

Landscape function is the capacity of a landscape to sustain energy flows, material cycling and information exchanges. It is regulated by the interactions between landscape pattern and ecological processes among landscape units [15–17], which change across temporal and spatial scales [18,19]. The concept of landscape function is similar to ecosystem service from a socio-economic perspective in that it seeks to characterize the capacity of a landscape to provide goods and services for human well-being directly or indirectly [15,20]. In this context, the diversity of landscape functions (i.e. landscape multi-functionality) has gathered recent attention for multi-disciplinary research [8,21,22]. Currently, research on landscape multi-functionality is widely recognized as a significant basis for sustainable land development [7,23,24].

Multi-functional landscape research and CZ science share common characteristics in their consideration of multiple, coincident and simultaneous functionalities on provisioning, regulating and supporting environmental goods and services. But contemporary multi-functional landscape research has in many cases failed to consider the underlying mechanisms of multi-functionality, such as driving forces and processes. While CZ science places much attention on structures, processes and mechanisms, no significant progress has been made on functions [14]. Therefore, the integration of multi-functional landscape research and CZ science can build a bridge for advancing both functional-oriented Earth science disciplines so that the challenges of sustainable development from local to global scales are met. The aim of multi-disciplinary integration is not to create new knowledge per se, but rather to solve complex problems that already exist, and thus create a new knowledge space for scientific progress [25]. This integration promotes the multi-disciplinarity of surface Earth system science as one of the key pillars for supporting sustainable development strategies [26].

This paper reviews scientific progress in these areas and discusses the emerging opportunities for synergistically advancing multi-functional landscape research and CZ science along a common functional perspective. The objectives of this paper are to: (i) review recent researches on multi-functional landscapes and CZ science; (ii) identify similarities, gaps and challenges of these two scientific disciplines; and (iii) propose strategies for multi-disciplinary and function-oriented research in support of sustainable CZs and landscapes.

## RESEARCH STATUS OF LANDSCAPE MULTI-FUNCTIONALITY AND CZ SCIENCE

### Multi-functional landscape

The concept of the multi-functional landscape was first proposed at the International Conference of Multi-functional Landscapes in Roskilde, Denmark in October 2000 [27]. In recent years, multi-functional landscape research has developed into an important field of landscape science, from theory and quantitative assessment to strategies in support of planning and management [7,11,15,22,28]. Conceptually, self-organization, non-equilibrium, dynamic evolution and hierarchy have all been identified as providing an important theoretical grounding for multi-dimensional and integrative landscape studies from a functional perspective [21]. To accurately define multi-functional landscapes, three criteria have been established [29]: (i) spatial independence—the spatial combination of functions associated with independent land units; (ii) periods of temporal independence—with respect to the plural, alternative and coincident multi-functionality of the same land unit at different times; and (iii) the spatial integration of functions—at the same or different periods, on the same or different land units. Research has ranged from initial multi-functional agroecosystems to forest and urban landscapes on topics considering the effects of the changing agricultural landscape structure on decision-making and the generation of public goods and services [15,30], forest landscape management and optimization [31,32] and the functionalities of green infrastructure, its planning and management [33–36].

In multi-functional landscape research, ecosystem services and landscape indices are generally used as substitute or proxy indicators of landscape functions supported by spatial and temporal statistical analyses. For example, timber production, carbon sequestration, landslides and erosion control have been used as indicators to compare the impacts of managed and unmanaged forest management regimes on forest landscape multi-functionality [31]. Methods based on landscape indices have also been proposed for multi-functional agroecosystems planning and design [37]. Other work has proposed the use of multiple ecosystem services landscape indices as tools to identify the drivers of functional landscape change and to quantify the conservation of landscape multi-functionality [38]. However, because of the lack of mechanisms to support index-based methods, together with an insufficient consideration of spatial heterogeneity and flows of ecosystem services, these methods are often difficult to apply in practice, such as to support landscape management, due to the large inferential uncertainties associated with measurement, metrics and the generalizability of the methods.

To quantify multiple landscape functions and their interactions, complex simulation models based on ecosystem services can be used. The InVEST (Integrated Valuation of Ecosystem Services and Trade-offs) model, based on a GIS platform, is a multi-module, multi-level, multi-scale and multi-scene analysis tool that allows ecosystem services to be quantified and mapped [39–41]. InVEST is widely used all over the world [42]. Other models have been developed for different purposes and approaches. For example, ARIES (ARtificial Intelligence for Ecosystem Services) emphasizes the actual physical flows of ecosystem services by networked models of provision, source and sink [40]. SolVES (Social Values for Ecosystem Services) focuses on biodiversity, aesthetic, cultural and economic values [43]. MIMES (Multi-scale Integrated Model of Ecosystem Services) simulates interactions of the natural and the human system [44], based on GUMBO (Global Unified Meta-model of the Biosphere) [42]. Of these models, the most widely applied is InVEST. In general, however, contemporary simulation models are loosely connected rather than closely coupled, which remains a methodological challenge for advancing quantitative multi-functional landscape research.

### CZ science

By definition, a CZ is a structured Earth surface entity that extends from the top of the vegetation canopy to the bottom of the groundwater aquifer and accommodates various biological, hydrological and geochemical processes. The CZ can be conceptualized as an open thermodynamic system through which the flux of energy and mass flow [45]. Quantifying the relevant influx of energy and mass under a theoretical framework of Environmental Energy and Mass Transfer (EEMT) provides a quantitative context for testing hypotheses about process coupling in the CZ across temporal and spatial scales [46,47]. Such frameworks have been used to quantify the relevant flux-gradient relations and the simulation of CZ evolution [48]. Research has suggested that EEMT is effective in predicting water-transit times, solution concentrations and mineral weathering processes [49]. Advances to this framework have a great potential to support the robust analysis and quantification of CZ functions and services.

The *criticality* of CZ science ultimately lies in its consideration of the inter-linked functionality that sustains human society. Therefore, CZ science requires an integrative orientation towards process and function beyond basic structural dimensions [50]. Process orientation emphasizes the complex dynamic processes and mechanisms of multi-element, multi-sphere and multi-scale research in CZ environments [51–53]. Functional orientation emphasizes important functions of CZs (e.g. environmental regulation, life support and resource supply) that are indispensable for the sustainable development of our human society. Studies that are functionally oriented are less common than those that are process-oriented.

The establishment of a series of CZ observatories has been the most significant achievement of CZ science since its inception [54–56]. To date, 62 CZ or CZ-like observatories have been established globally, with the majority in North America and Europe (http://wiki.seg.org/wiki/Critical_zone (31 January, 2018, date last accessed)). Much of the work of CZOs has considered the biological and geophysical structural aspects of the CZ [46–48]. Some recent research has focused on the fundamental laws of the formation and evolution of CZs and observations of their complex structures and processes [57]. CZ process-oriented studies have examined water cycling, nutrient and material transport represented mainly by land-atmospheric conversion of carbon and water, soil moisture content, pore water chemistry, transformation of surface water, soil water and groundwater, soil long-term evolution and other processes [49,56–61].

Research on the functions and services associated with the CZ are still at the theoretical stage. Improving the understanding on CZ functions is important for predicting their sensitivity to complex environmental changes and for devising adaptive management responses in the Earth surface system [14]. Theoretically, research on and consideration of CZ services (i.e. the subset of CZ functions that are recognized as beneficial to human society) are able to provide context, constraints and a currency for understanding and quantifying ecosystem services, thus providing valuable support for decision-making [2]. Measurement and mathematical modeling have also been advocated to advance integrative knowledge, particularly to link soil structure to soil processes [62] and CZ services [63]. Despite these initiatives, the ecosystem service as a scientific pursuit is somewhat primitive, both conceptually and methodologically, and integrative methodologies that combine ecosystem service concepts and methodologies with CZ science are only just emerging.

## COMMON PROPERTIES OF MULTI-FUNCTIONAL LANDSCAPE RESEARCH AND CZ SCIENCE AS A BASIS FOR INTEGRATION

### Spatial heterogeneity

Landscape heterogeneity in the horizontal dimension reflects the inherent spatial complexity of landscape structure, which in turn presents a significant difficulty in understanding landscape processes. For example, landscape heterogeneity affects the flow and spread of resources, species and disturbances, which has important implications on the regulation of landscape functions [17]. Horizontally, landscape functions are dependent on geographical location and scale, whilst simultaneously the dynamics of landscape function can vary along temporal scales—from years to decades to centuries. These spatiotemporal features need to be fully considered for the robust assessment, planning and management of multi-functional landscapes. Understanding and quantifying landscape heterogeneity support reliable assessments of natural and social landscape characteristics for predicting biodiversity [64], increasing ecosystem function and resilience [65] and implementing landscape planning [34].

The CZ is also a spatially heterogeneous entity [57,66] whose horizontal heterogeneities can be summarized via three aspects [57,67]: (i) internal factors related to geology and hydrology; (ii) external factors related to climate and natural fires; and (iii) human factors related to land use, urbanization and other activities. CZ horizontal heterogeneities influence surface processes and functions [48], in a similar way to landscapes. However, vertical heterogeneity along the deep depth of a CZ profile has been considered to be of greater importance than horizontal heterogeneity [14], providing a clear point of difference with respect to spatial heterogeneity between CZ science and landscape research.

### Continuous evolution

The impacts of human forcing are increasingly recognized alongside the forcing of tectonics, weathering, fluid transport and biological activities, as highlighted by the NRC (2001) [1]. Land-use changes are anthropogenically driven by change in societal need, which in turn drives changes in the CZ and the landscape [28] and critically changes the nature and importance of landscape multi-functionality. Changes in the CZ are generally irreversible and cumulative, where human alterations to the CZ have also become pervasive and long-lasting [1]. This is because of the coupling of complex physical, chemical and biological processes that drive the dynamics of the CZ [1,57]. For example, soil thickness in the CZ is gradually reducing and the reduction rate is several times that of soil formation [55]. Actual soil-erosion rates could be accelerated significantly by human disturbances (e.g. via deforestation and hill-slope farming) compared to natural soil-loss rates that may lead to environmental problems such as land degradation and diffuse pollution. This continuous, inseparable and constantly changing system has a number of common features at both landscape and CZ scales.

### Close relationships with ecosystem services

Ecosystem services are the benefits that humans acquire from ecosystems. These have been classified into four general categories: (i) provisioning services, (ii) regulating services, (iii) supporting services and (iv) cultural services [68]. The categories emphasize the importance of ecosystems in supporting human social systems, and provide a theoretical basis for understanding ecosystem function and value that promotes the protection of ecosystems and implementing strategies for their restoration and sustainable development.

Landscape functions are embodied in landscape structures as well as the mosaic of ecosystem processes and functions. The identification and quantification of landscape multi-functionality are often associated with trade-off, synergies and the integration of ecosystem services [38]. Ecosystem services are thus often regarded as the key component of representing the landscape functions in the conceptualization of multi-functional landscapes [38]. Landscape functions are frequently divided into four types [7,23] relating to: (i) production functions, (ii) regulating function, (ii) habitat function and (iv) information functions. Ecosystem services provide the basic elements for quantifying landscape multi-functionality and for describing the hierarchical relationships between ecosystems and landscapes.

CZ services and associated processes are conceptually correlated with the demand of human society from a functional perspective, in which ecosystem services constitute an important part, and simultaneously research into an ecosystem service can be supported by considering the context-constant-currency CZ service framework [2]. Therefore, sustainable management of the CZ for human society in the face of environmental stress requires a holistic understanding of CZ services. As a practical example, the SoilTrEC project quantified ecosystem services in the CZ and the effects of environmental change on key soil functions, together with providing decision-support tools based on research results integration [55,63,69].

The above three themes and properties relating to spatial heterogeneity, continuous evolution and ecosystem service are disciplinary considerations common to landscapes and CZs. These themes provide a basis for integrative researches to improve the scientific understanding of both landscapes and CZs as multi-functional Earth surface systems.

## ADVANCING CZ SCIENCE AND MULTI-FUNCTIONAL LANDSCAPE RESEARCH BY INTEGRATION

### Strengthening function-oriented CZ science

The importance of the CZ is reflected through both its natural and socio-economic functionality and would be strengthened through the integrative study of the functionality and multi-functionality of CZs at different spatiotemporal scales. Such function-oriented approaches can be promoted according to different categories of CZs as defined on their biophysical or management heterogeneities (e.g. agricultural CZs, urban CZs, conservation areas as CZs). Relevant research themes range from simple function identification and assessment to the consideration of the dynamics of CZ functions and associated driving forces, and then to strategies and models to optimize the sustainable management of CZ functions. Integrated methods for multi-functional quantitative landscape assessment, planning and management could be derived from an increased focus on function-oriented CZ science with appropriate considerations of *deep* depth, *deep* time and *deep* coupling [14], related to decision-making processes.

### Consolidating multi-functional landscape and CZ research by process-based mechanistic analyses

Contemporary CZ science has found strong support from structural, processes and evolutional perspectives. What is currently weak is an integrative understanding of the CZ multi-functionality through critical representations of how CZ structures and processes interact across spatiotemporal scales. Multi-functional landscape research in this area has made significant progress over the last decade, which has the potential to strengthen the adoption of a process-supported CZ framework to facilitate process-based and mechanistic landscape functionality studies. Processes may include, but are not limited to, the hydrologic cycle, the geochemical cycle, the carbon cycle, the nutrient cycle, gaseous exchange, erosion and deposition, weathering, soil formation and evolution, life processes and human impacts. This can lead multi-functional landscape research to a more scientifically robust stage, enabling more informed and powerful decision support to landscape planning and management.

**Figure 1.**
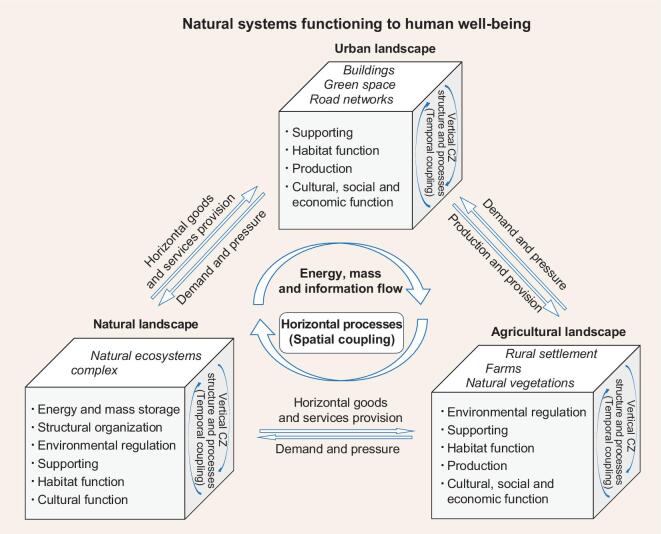
Diagrammatic layout of deep coupling between CZs and landscapes. In which, each cube indicates a type of landscapes, including natural, agricultural and urban landscapes. The top of the cube indicates main elements contained in the landscape. The front of the cube indicates CZ functions of the landscape. Linear arrows indicate the dominant relationship between them. Circular arrows indicate processes.

The multi-functionality of CZs can also be considered through an integrative landscape perspective (Fig. 1). The coupling of horizontal and vertical processes integrates the multi-functionality of CZs and landscapes. In the vertical direction, coupling links water and nutrition transfers with weathering processes in the CZs (from bedrock to soil and vegetation) across time scales that range from seconds to millions of years. For multi-functional landscape management, processes characterized by seasonal, annual and decadal time scales need particular consideration, while other processes provide context or background considerations that directly support decision-making. Likewise, process-based mechanistic analyses can also promote spatiotemporal scaling and coupling [70].

The sustainable use and management of landscapes emphasized by CZ science provide strong scientific support through consideration of the above processes and their coupling effects. Function-oriented landscape development and conservation decisions, based on advanced process-based studies, can inform and facilitate landscape sustainability. In the horizontal direction, coupling considers the spatial flows of material, energy, information and landscape services. Among the three types of landscapes shown in Fig. 1, urban landscapes depend largely on agricultural landscapes for food and fiber, with natural landscapes pervasive to all landscapes and underpinning environmental quality. Therefore, the multi-dimensional integration of multi-functional landscape research and CZ science can contribute to a concerted science-based resolution of environmental degradation and pollution problems, as a prerequisite to sustainable development in Earth surface systems.

### Global alliance for monitoring

Both CZ science and multi-functional landscape research cannot develop without consideration of structural, process and functional perspectives. Ecological monitoring provides the basis of ecosystem services and the integrated assessment of landscape functions. The diversity of the ecosystem services and landscape multi-functionality requires a multi-dimensional approach to ecological monitoring from both field-based small-scale measures and multi-resolution remote-sensing-based monitoring [71,72]. There are many monitoring systems for ecosystems and landscapes, such as the International Long Term Ecological Research (ILTER) [73], the Global Environment Monitoring System (GEMS) [74], the Global Terrestrial Observing System (GTOS) [75], the Chinese Ecosystem Research Network (CERN) [76,77], the Long Term Ecological Research (LTER) in the USA [78], the Terrestrial Ecosystem Research Network (TERN) in Australia [65,79] and the Environmental Change Monitoring Network (ECN) in the UK [80]. Importantly, from a global perspective, the development of an international monitoring network of CZs is needed.

Integrating CZ observatories into a global network broadens our understanding of processes at larger spatial scales, providing deeper insights and advancing our understanding of the integration and coupling of Earth surface processes [25]. Therefore, current monitoring facilities across the globe tend to be networked and are multi-disciplinary. Other monitoring networks also have the potential to be integrated through expanding their multi-disciplinarity (towards trans-disciplinarity) and can be more resource-efficient than merely establishing new monitoring sites for a single scientific purpose. New monitoring sites may be needed if the current configuration of current sites is found to be insufficient to represent a major CZ or global land-surface landscape type, suggesting the need for on-going reviews of monitoring sites and their potential relocation. This suggests the need for and promotion of close collaboration among the existing monitoring whilst simultaneously reviewing and planning the requirements for representative monitoring sites at both local and global scales.

### Scaling and coupling by modeling

Landscape and CZs are multi-scale hierarchical systems with common spatial heterogeneity and temporal dynamics. They require spatiotemporal scaling and the coupled modeling of complex interacting processes for improved understanding and management of Earth surface systems. Practical solutions for temporal scaling lie in a suitable coupling and integration of different processes together with an understanding of various drivers of change, and their temporal scales, in CZs and multi-functional landscapes. For example, coupling of biogeochemical and hydropedological processes has been investigated at Boulder Creek CZ observatory in Colorado [81], the Catalina-Jemez CZ observatory [48] and the Shale Hills CZ observatory [82]. Research has shown that interactions exist between fast cyclic processes (e.g. diurnal fluctuation of soil moisture and yearly changes in vegetation growth) and long-term cumulative changes (e.g. bedrock weathering, pedogenesis and ecosystem succession) [83]. Besides the coupling of biogeochemical and hydropedological processes, other processes need to be further investigated, especially across different temporal scales [14].

Spatial scales can be qualitatively categorized into three relevant domains of micro-scale, meso-scale and macro-scale. At present, CZ monitoring includes two of these categories: one uses (ground-based) sensor technology to monitor at the micro-scale, whilst the other uses remote sensing technology to monitor at the macro-scale. The technology between the two scales is still immature, leaving much scope for development. The inconsistency between the scale of the proves being observed and the monitoring (or observational) scale is a challenge for process and modeling research and, as a consequence, scaling is an important issue for CZ and landscape sciences [57,84]. However, the objective of (down- or up-) scaling is to reveal the interactions between patterns and processes operating within the hierarchical landscape and the CZ systems, which are often highly non-linear and dynamic [85]. According to the hierarchical theory of O’Neill *et al.* [86], each scale has its own constraints and thresholds, so it can be difficult apply the same constraints and thresholds across scales when scaling up or down. Similarly, there are large uncertainties for down-scaling from the whole landscape to the ecosystem or to the pedon [11]. Research often follows an integrative multiple-scale approach that establishes a set of rules and algorithms in the modeling system for scaling. Research at the micro-scale has found that up-scaling to the macro-scale can provide a comprehensive analysis of regional ecosystem services and landscape functions. For example, an Australian research team achieved a carbon and water balance with 1-km resolution by the coupling of an ecosystem model and a meso-scale model. Up-scaling to the regional level was supported by airborne remote sensing methods, before down-scaling to the site and leaf level [87]. A multi-scale analysis framework has also been established for the dynamic simulation of landscape functions, with consideration of the local scale, the management scale and the regional scale [88].

In the vertical direction, coupling includes two categories. One links above-ground systems to below-ground systems and the other one links the shallow root zone soil to deep weathered bedrock [14]. The former has attracted much attention through multi-disciplinary and trans-disciplinary studies due to the cross-scale consideration of land–atmosphere interactions. The latter requires further investigation of more advanced monitoring and modeling because surface soil cycles operate at small spatiotemporal scales, while the deeper groundwater and weathered bedrock cycles operate at much larger spatiotemporal scales.

Clearly, scaling and systematic coupling can be addressed by modeling. Multi-functional landscape and CZ systems have inherent sensitivities in responses to land-use changes [14,22]. Thus, multi-functional landscape and CZ model simulations depend strongly on land-use changes, but also on land-cover change and knowledge of multiple other processes [14,22]. There are three ways of model coupling. First, the models are related to different processes, such as biogeochemical and hydropedological processes, as mentioned above [14]. Other processes can also be monitored for coupling [89], such as hydrologic processes with sediment-transport processes [90,91], using the multi-component Reactive Transport Models (RTMs) [92]. Coupling models should include links between pedogenesis and landscape evolution [93] and between anthropogenic and natural processes. Second, models can be coupled multi-dimensionally. Processes in CZs are multi-directional, so multi-dimensional mapping is an important technology for predicting the heterogeneous structures and processes in CZs and multi-functional landscapes, as Earth surface systems. Third, the coupling of conceptual and methodological models needs to be directed by a systematic framework for more effective real-world problem-solving. Conceptual and methodological models are used to investigate important flows (e.g. water, energy, solutes, carbon, nitrogen and sediment) and to quantify the distribution of topographical and environmental features [94], which cannot be addressed by any single model, separately.

## CONCLUSION

Multi-functional landscape research and CZ science are two emerging fields in Earth system science. This paper reviews research progress and the commonalities of the two scientific disciplines, as a first step for their potential integration. Each paradigm emphasizes continuous evolution and a high degree of process heterogeneity in both horizontal and vertical directions and maintaining a close relationship with ecosystem services. Based on these commonalities, this paper suggests a number of potential advances through the integration of different strands of multi-functional landscape research with CZ science, by strengthening function-oriented CZ science, process-based mechanistic analyses for multi-functional landscapes, global long-term and networked monitoring, and systematic modeling supported by scaling and deep coupling. Multi-disciplinary integration can support the advancement of both function-oriented landscape and CZ research in order to meet future planning and management needs at a variety of spatiotemporal scales. This is a key challenge for sustainable development and human well-being in the twenty-first century.

## References

[1] Lin H. Earth's Critical Zone and hydropedology: concepts, characteristics, and advances. Hydrol Earth Syst Sci 2010; 14: 25–45. 10.5194/hess-14-25-2010

[2] Field JP , Breshears DD, Law DJ et al. Critical Zone services: expanding context, constraints, and currency beyond ecosystem services. Vadose Zone J 2015; 14: 7. 10.2136/vzj2014.10.0142

[3] Brantley SL , DiBiase RA, Russo TA et al. Designing a suite of measurements to understand the critical zone. Earth Surf Dynam 2016; 4: 211–35. 10.5194/esurf-4-211-2016

[4] Anderson SP , von Blanckenburg F, White AF. Physical and chemical controls on the Critical Zone. Elements 2007; 3: 315–9. 10.2113/gselements.3.5.315

[5] O’Neill RV . Is it time to bury the ecosystem concept? (With full military honors of course!). Ecology 2001; 82: 3275–84.

[6] Richter DD , Billings SA. ‘One physical system’: Tansley's ecosystem as Earth's critical zone. New Phytol 2015; 206: 900–12. 10.1111/nph.13338 25731586

[7] de Groot R. Function-analysis and valuation as a tool to assess land use conflicts in planning for sustainable, multi-functional landscapes. Landscape Urban Plan 2006; 75: 175–86. 10.1016/j.landurbplan.2005.02.016

[8] Fry GLA. Multifunctional landscapes—towards transdisciplinary research. Landscape Urban Plan 2001; 57: 159–68. 10.1016/S0169-2046(01)00201-8

[9] O’Farrell PJ , Anderson PML. Sustainable multifunctional landscapes: a review to implementation. Curr Opin Env Sust 2010; 2: 59–65. 10.1016/j.cosust.2010.02.005

[10] O’Farrell PJ , Reyers B, Le Maitre DC et al. Multi-functional landscapes in semi arid environments: implications for biodiversity and ecosystem services. Landscape Ecol 2010; 25: 1231–46. 10.1007/s10980-010-9495-9

[11] Reyers B , O’Farrell PJ, Nel JL et al. Expanding the conservation toolbox: conservation planning of multifunctional landscapes. Landscape Ecol 2012; 27: 1121–34. 10.1007/s10980-012-9761-0

[12] Jiao YM , Xiao DN, Guo M. Landscape and the holistic research in landscape ecology. (Chinese) Geog Geo Info Sci 2003; 1: 91–5.

[13] Xiao DN , Li XZ, Gao J et al. Landscape ecology [M]. (Chinese) Beijing: Science Press, 2003.

[14] Guo L , Lin H. Critical zone research and observatories: current status and future perspectives. Vadose Zone J 2016; 15: 1–14. 10.2136/vzj2016.06.0050

[15] Brown I , Castellazzi M. Scenario analysis for regional decision-making on sustainable multifunctional land uses. Reg Environ Change 2014; 14: 1357–71. 10.1007/s10113-013-0579-3

[16] Lovell ST , DeSantis S, Nathan CA et al. Integrating agroecology and landscape multifunctionality in Vermont: an evolving framework to evaluate the design of agroecosystems. Agr Syst 2010; 103: 327–41. 10.1016/j.agsy.2010.03.003

[17] Fu BJ , Chen LD, Ma KM et al. Theory and application of landscape ecology [M]. (Chinese) Beijing: Science Press, 2011.

[18] Openshaw S. Ecological fallacies and the analysis of areal census data. Environ Plan A 1984a; 16: 17–31. 10.1068/a160017 12265900

[19] Openshaw S . Concepts and Techniques in Modern Geography 38: The Modifiable Areal Unit Problem. Geo Books, Norwich 1984.

[20] Willemen L , Hein L, van Mensvoort MEF et al. Space for people, plants, and livestock? Quantifying interactions among multiple landscape functions in a Dutch rural region. Ecol Indic 2010; 10: 62–73. 10.1016/j.ecolind.2009.02.015

[21] Naveh Z . Ten major premises for a holistic conception of multifunctional landscapes. Landscape Urban Plan 2001; 57: 269–84. 10.1016/S0169-2046(01)00209-2

[22] Peng J , Lv HL, Liu YX et al. International research progress and perspectives on multfunctional landscape. (Chinese) Adv Earth Sci 2015; 30: 465–76.

[23] Lovell ST , Johnston DM. Creating multifunctional landscapes: how can the field of ecology inform the design of the landscape? Front Ecol Environ 2009; 7: 212–20. 10.1890/070178

[24] Villamor GB , van Noordwijk M, Djanibekov U et al. Gender differences in land-use decisions: shaping multifunctional landscapes? Curr Opin Env Sust 2014; 6: 128–33. 10.1016/j.cosust.2013.11.015

[25] Anderson SP , Bales RC, Duffy CJ. Critical Zone observatories: building a network to advance interdisciplinary study of Earth surface processes. Mineral Mag 2008; 72: 7–10. 10.1180/minmag.2008.072.1.7

[26] Lin H , Hopmans JW, Richter DD. Interdisciplinary sciences in a global network of Critical Zone observatories. Vadose Zone J 2011; 10: 781–5. 10.2136/vzj2011.0084

[27] Brandt J. Multifunctional landscapes—perspectives for the future. J Environ Sci 2003; 15: 187–92. 12765260

[28] Tang Q , Ding SY. A review of multifunctional landscape. (Chinese) Acta Ecol Sin 2014; 36: 3151–7.

[29] Zhou HR . Prospect on multifunctional landscape of marshes in arid areas. (Chinese) Arid Land Geog 2005; 28: 16–20.

[30] Harden NM , Ashwood LL, Bland WL et al. For the public good: weaving a multifunctional landscape in the Corn Belt. Agric Hum Values 2013; 30: 525–37. 10.1007/s10460-013-9429-7

[31] Irauschek F , Rammer W, Lexer MJ. Can current management maintain forest landscape multifunctionality in the Eastern Alps in Austria under climate change? Reg Envir Chang 2015; 17: 1–16.

[32] Santika T , Meijaard E, Wilson KA. Designing multifunctional landscapes for forest conservation. Environ Res Lett 2015; 10: 9. 10.1088/1748-9326/10/11/114012

[33] Hansen R , Pauleit S. From multifunctionality to multiple ecosystem services? A conceptual framework for multifunctionality in green infrastructure planning for urban areas. AMBIO 2014; 43: 516–29. 10.1007/s13280-014-0510-2 24740622 PMC3989511

[34] Lovell ST , Taylor JR. Supplying urban ecosystem services through multifunctional green infrastructure in the United States. Landscape Ecol 2013; 28: 1447–63. 10.1007/s10980-013-9912-y

[35] Schindler S , Sebesvari Z, Damm C et al. Multifunctionality of floodplain landscapes: relating management options to ecosystem services. Landscape Ecol 2014; 29: 229–44. 10.1007/s10980-014-9989-y

[36] Stockdale A , Barker A. Sustainability and the multifunctional landscape: an assessment of approaches to planning and management in the Cairngorms National Park. Land Use Policy 2009; 26: 479–92. 10.1016/j.landusepol.2008.07.001

[37] Lovell ST , Mendez VE, Erickson DL et al. Extent, pattern, and multifunctionality of treed habitats on farms in Vermont, USA. Agroforest Syst 2010; 80: 153–71. 10.1007/s10457-010-9328-5

[38] Rodriguez-Loinaz G , Alday JG, Onaindia M. Multiple ecosystem services landscape index: a tool for multifunctional landscapes conservation. J Environ Manage 2015; 147: 152–63. 10.1016/j.jenvman.2014.09.001 25265555

[39] Vigerstol KL , Aukema JE. A comparison of tools for modeling freshwater ecosystem services. J Environ Manage 2011; 92: 2403–9. 10.1016/j.jenvman.2011.06.040 21763063

[40] Wu Z , Chen X, Liu BB et al. Research progress and application of InVEST model. (Chinese) Chin J Tropical Agri 2013; 33:58–62.

[41] Yang YY , Dai EF, Fu H. The assessment framework of ecosystem service value based on InVEST model. (Chinese) J Capital Normal Univ (Natural Science Edition) 2012; 33: 41–7.

[42] Huang CH , Yang J, Zhang WJ. Development of ecosystem services evaluation models: research progress. (Chinese) Chin J Ecol 2013; 32: 3360–7.

[43] Sherrouse BC , Semmens DJ, Clement JM. An application of Social Values for Ecosystem Services (SolVES) to three national forests in Colorado and Wyoming. Ecol Indic 2014; 36: 68–79. 10.1016/j.ecolind.2013.07.008

[44] Boumans R , Roman J, Altman I et al. The Multiscale Integrated Model of Ecosystem Services (MIMES): simulating the interactions of coupled human and natural systems. Ecosyst Serv 2015; 12: 30–41. 10.1016/j.ecoser.2015.01.004

[45] Rasmussen C , Pelletier JD, Troch PA et al. Quantifying topographic and vegetation effects on the transfer of energy and mass to the Critical Zone. Vadose Zone J 2015; doi: 10.2136/vzj2014.07.0102. 10.2136/vzj2014.07.0102

[46] Rasmussen C , Troch PA, Chorover J et al. An open system framework for integrating critical zone structure and function. Biogeochemistry 2011; 102: 15–29. 10.1007/s10533-010-9476-8

[47] Zapata-Rios X , Brooks PD, Troch PA et al. Influence of climate variability on water partitioning and effective energy and mass transfer in a semi-arid critical zone. Hydrol Earth Syst Sci 2016; 20: 1103–15. 10.5194/hess-20-1103-2016

[48] Chorover J , Troch PA, Rasmussen C et al. How water, carbon, and energy drive critical zone evolution: the Jemez–Santa Catalina Critical Zone Observatory. Vadose Zone J 2011; 10: 884–99. 10.2136/vzj2010.0132

[49] Zapata-Rios X , McIntosh J, Rademacher L et al. Climatic and landscape controls on water transit times and silicate mineral weathering in the critical zone. Water Resour Res 2015; 51: 6036–51. 10.1002/2015WR017018

[50] Connor JD , Bryan BA, Nolan M et al. Modelling Australian land use competition and ecosystem services with food price feedbacks at high spatial resolution. Environ Modell Softw 2015; 69: 141–54. 10.1016/j.envsoft.2015.03.015

[51] Ding YJ , Zhang SQ, Han TD et al. Opportunities and challenges of studies across land surface processes to land surface system sciences [J]. (Chinese) Adv Earth Sci 2014; 29: 443–55.

[52] Li XY . Coupling, response and adaptation mechanisms of soil-vegetation-hydrology in arid areas. (Chinese) Scientia Sinica (Terrae) 2011; 41: 1721–30.

[53] Yu GR , Gao Y, Wang QF et al. Discussion on the key processes of carbon-nitrogen-water coupling cycles and biological regulation mechanisms in terrestrial ecosystem. (Chinese) Chin J Eco Agri 2013; 21: 683–98.

[54] Anderson SP , Anderson RS, Tucker GE. Landscape scale linkages in critical zone evolution. C R Geosci 2012; 344: 586–96. 10.1016/j.crte.2012.10.008

[55] Banwart S , Bernasconi SM, Bloem J et al. Soil processes and functions in Critical Zone observatories: hypotheses and experimental design. Vadose Zone J 2011; 10: 974–87. 10.2136/vzj2010.0136

[56] Cheng GD , Li X, Zhao WZ et al. Integrated study of the water–ecosystem–economy in the Heihe River Basin. Natl Sci Rev 2014; 1: 413–28. 10.1093/nsr/nwu017

[57] Yang JF , Zhang CG. Critical Zone: a new framework of Geological environment research. (Chinese) Hydrogeo Engine Geol 2014: 98–104.

[58] Bagstad KJ , Semmens D, Winthrop R et al. Ecosystem services valuation to support decisionmaking on public lands: a case study of the San Pedro River watershed, Arizona. U.S. Geological Survey Scientific Investigations Report 2012.

[59] Moraetis D , Paranychianakis NV, Nikolaidis NP et al. Sediment provenance, soil development, and carbon content in fluvial and manmade terraces at Koiliaris River Critical Zone Observatory. J Soils Sediments 2015; 15: 347–64. 10.1007/s11368-014-1030-1

[60] Stone MM , DeForest JL, Plante AF. Changes in extracellular enzyme activity and microbial community structure with soil depth at the Luquillo Critical Zone Observatory. Soil Biol Biochem 2014; 75: 237–47. 10.1016/j.soilbio.2014.04.017

[61] Stone MM , Kan JJ, Plante AF. Parent material and vegetation influence bacterial community structure and nitrogen functional genes along deep tropical soil profiles at the Luquillo Critical Zone Observatory. Soil Biol Biochem 2015; 80: 273–82. 10.1016/j.soilbio.2014.10.019

[62] Bui EN. Data-driven Critical Zone science: a new paradigm. Sci Total Environ 2016; 568: 587–93. 10.1016/j.scitotenv.2016.01.202 26883371

[63] Banwart S , Menon M, Bernasconi SM et al. Soil processes and functions across an international network of Critical Zone observatories: introduction to experimental methods and initial results. C R Geosci 2012; 344: 758–72. 10.1016/j.crte.2012.10.007

[64] Jorgensen A , Gobster P. Shades of green: measuring the ecology of urban green space in the context of human health and well-being. Nature & Culture 2010; 5: 338–63. 10.3167/nc.2010.050307

[65] Fischer J , Lindenmayer DB, Manning AD. Biodiversity, ecosystem function, and resilience: ten guiding principles for commodity production landscapes. Front Ecol Environ 2006; 4: 80–6. 10.1890/1540-9295(2006)004[0080:BEFART]2.0.CO;2

[66] Lin H , Bouma J, Wilding LP et al. Advances in hydropedology. In: Sparks DL (ed.). Advances in Agronomy, Vol. 85. San Diego: Elsevier Academic Press Inc., 2005, 1–89.

[67] Bell S. Landscape: Patterns, Perception and Process. London: E & FN Spon, 1999.

[68] Li SC , Liu JL, Zhang CY et al. The research trends of ecosystem services and the paradigm in geography. (Chinese) Acta Geograph Sin 2011; 66: 1618–30.

[69] Menon M , Rousseva S, Nikolaidis NP et al. SoilTrEC: a global initiative on critical zone research and integration. Environ Sci Pollut Res 2014; 21: 3191–5. 10.1007/s11356-013-2346-x 24310904

[70] Riebe CS , Hahm WJ, Brantley SL. Controls on deep critical zone architecture: a historical review and four testable hypotheses. Earth Surf Process Landforms 2017; 42: 128–56. 10.1002/esp.4052

[71] Sommerville MM , Milner-Gulland EJ, Jones JPG. The challenge of monitoring biodiversity in payment for environmental service interventions. Biol Conserv 2011; 144: 2832–41. 10.1016/j.biocon.2011.07.036

[72] Lv YH , Ma ZM, Fu BJ et al. Diversity of ecosystem services and landscape multi-functionality: from scientific concepts to integrative assessment. (Chinese) Acta Ecol Sin 2013; 33: 1153–9. 10.5846/stxb201204130533

[73] Maass M , Equihua M. La Red Internacional de Investigación Ecológica a Largo Plazo (ILTER) a 20 años de su creación: sus avances y retos. Bosque (Valdivia) 2014; 35: 415–9. 10.4067/S0717-92002014000300016

[74] Liu HJ , Sun C, Qi Y et al. A brief review of progress of the eco-environmental research network in China and abroad. (Chinese) Environm Monitor China 2014; 30:125–31.

[75] Kondratev KY . Global Terrestrial Observing System (GTOS)—detection and monitoring of continental ecosystems. Earth Obs Remot Sen 1995; 12: 468–73.

[76] Zhao SD . Chinese Ecosystem Research Network (CERN): introduction and progress. Ambio 1999; 28: 636–8.

[77] Fu BJ , Lv YH, Chen LD et al. The latest progress of landscape ecology in the world. (Chinese) Acta Ecol Sin 2008; 02: 798–804.

[78] Redman CL , Grove JM, Kuby LH. Integrating social science into the long-term ecological research (LTER) network: social dimensions of ecological change and ecological dimensions of social change. Ecosystems 2004; 7: 161–71. 10.1007/s10021-003-0215-z

[79] Lynch AJJ , Thackway R, Specht A et al. Transdisciplinary synthesis for ecosystem science, policy and management: the Australian experience. Sci Total Environ 2015; 534: 173–84. 10.1016/j.scitotenv.2015.04.100 25957785

[80] Morecroft MD , Bealey CE, Beaumont DA et al. The UK Environmental Change Network: emerging trends in the composition of plant and animal communities and the physical environment. Biol Conserv 2009; 142: 2814–32. 10.1016/j.biocon.2009.07.004

[81] Dethier DP , Bove DJ. Mineralogic and geochemical changes from alteration of granitic rocks, Boulder Creek Catchment, Colorado. Vadose Zone J 2011; 10: 858–66. 10.2136/vzj2010.0106

[82] Jin LX , Andrews DM, Holmes GH et al. Opening the ‘black box’: water chemistry reveals hydrological controls on weathering in the Susquehanna Shale Hills Critical Zone Observatory. Vadose Zone J 2011; 10: 928–42. 10.2136/vzj2010.0133

[83] Lin H . A new worldview of soils.Soil Sci Soc Am J 2014; 78: 1831–44. 10.2136/sssaj2014.04.0162

[84] Xu ZY , Hu YF, Liu Y et al. A review on the accuracy analysis of spatial scaling data. (Chinese) Prog Geog 2012; 31: 1574–82.

[85] Lai YC . Controlling complex, non-linear dynamical networks. Natl Sci Rev 2014; 1: 339–41. 10.1093/nsr/nwu023

[86] O'Neill RV , DeAngelis DL, Waide JB et al. A Hierarchical Concept of Ecosystems. Princeton: Princeton University of Press, 1986.

[87] Beringer J , Hutley LB, Hacker JM et al. Patterns and processes of carbon, water and energy cycles across northern Australian landscapes: from point to region. Agr Forest Meteorol 2011; 151: 1409–16. 10.1016/j.agrformet.2011.05.003

[88] Willemen L , Veldkamp A, Verburg PH et al. A multi-scale modelling approach for analysing landscape service dynamics. J Environ Manage 2012; 100: 86–95. 10.1016/j.jenvman.2012.01.022 22366361

[89] Pangle LA , DeLong SB, Abramson N et al. The Landscape Evolution Observatory: a large-scale controllable infrastructure to study coupled Earth-surface processes. Geomorphology 2015; 244: 190–203. 10.1016/j.geomorph.2015.01.020

[90] de Mello CR , Norton LD, Pinto LC et al. Agricultural watershed modeling: a review for hydrology and soil erosion processes. Ciênc agrotec 2016; 40: 7–25. 10.1590/S1413-70542016000100001

[91] Zhang Y , Slingerland R, Duffy C. Fully-coupled hydrologic processes for modeling landscape evolution. Environ Modell Softw 2016; 82: 89–107. 10.1016/j.envsoft.2016.04.014

[92] Li L , Maher K, Navarre-Sitchler A et al. Expanding the role of reactive transport models in critical zone processes. Earth-Sci Rev 2017; 165: 280–301. 10.1016/j.earscirev.2016.09.001

[93] Temme A , Vanwalleghem T. LORICA—a new model for linking landscape and soil profile evolution: development and sensitivity analysis. Comput Geosci 2016; 90: 131–43. 10.1016/j.cageo.2015.08.004

[94] Duffy C , Shi YN, Davis K et al. Designing a Suite of Models to Explore Critical Zone Function. In: Gaillardet J (ed.). Geochemistry of the Earth's Surface Ges-10. Amsterdam: Elsevier Science Bv, 2014, 7–15.

